# Case Report: *ALK*-rearranged mesenchymal neoplasms with S100 and CD34 co-expression: additional cases with distinct characteristics

**DOI:** 10.3389/fonc.2025.1516491

**Published:** 2025-05-29

**Authors:** Qi Ouyang, Xiaohong Guo, Rongjun Mao, Zhixing Cao

**Affiliations:** ^1^ Department of Pathology, Zhuhai People’s Hospital (Zhuhai Clinical Medical College of Jinan University), Zhuhai, Guangdong, China; ^2^ Department of Pathology, Foshan Hospital of Traditional Chinese Medicine, Foshan, Guangdong, China

**Keywords:** case report, cutaneous, spindle tumor, infantile fibrosarcoma-like tumor, *ALK* rearrangement

## Abstract

*ALK* rearrangements are rarely documented in superficial soft tissue neoplasms exhibiting an infantile fibrosarcoma-like spindle cell tumor (IFS) pattern or stromal, resembling Neurotrophic Tyrosine Kinase Receptor(*NTRK*)rearranged spindle cell tumors. Here, we present two cases of pediatric cutaneous soft tissue tumors with an IFS pattern, in which *ALK* fusions involving related partner genes were identified. The tumors in both cases demonstrated similar morphology and consisted of ovoid and spindle cells with infiltrative boundaries. The spindle cells exhibited either a fascicular growth pattern or a haphazard pattern and stromal hyalinization. Both cases involved inflammatory cell infiltration, brisk mitosis, and CD34, S100, and ALK-D5F3 immunoreactivity. Next-generation sequencing identified *ALK* fusion with different partner genes (*STRN* and *PLEKHH2*). The fluorescence *in situ* hybridization break-apart assay confirmed ALK rearrangements in both cases. In case 1, no indications of disease progression or metastasis was observed within the limited follow-up (36 months). However, the patient in case 2 experienced a rapid recurrence and metastasis.

## Introduction

The *ALK* gene on chromosome 2p23 encodes a receptor tyrosine kinase crucial for brain development and the functioning of certain nervous system neurons (1). *ALK* gene fusions are mutually exclusive oncogenic drivers and have been extensively documented in ALK-positive anaplastic large-cell lymphoma (ALCL), inflammatory myofibroblastic tumors (IMT) (2), non-small cell lung cancer (NSCLC), Spitz tumors, and Merkel cell carcinoma. *ALK*-rearranged soft tissue tumors were recently reported and involve various nomenclatures that have not been standardized. These include *ALK*-rearranged inflammatory myofibroblastic tumors (2), *ALK*-rearranged low-grade spindle cell tumor (3), superficial *ALK*-rearranged myxoid spindle cell tumors (4), *ALK*-rearranged infantile fibrosarcoma-like (IFS) tumors (5), *ALK*-rearranged histiocytosis (6), and *ALK*-rearranged cutaneous epithelioid fibrous histiocytomas (7). These tumors may exhibit benign, low-intermediate, or high-grade biological behaviors. *ALK*-rearranged low-grade spindle cell tumors share morphological and immunohistochemical features with *ALK*-rearranged IFS tumors without essential differences, indicating that the two tumors have highly identical low-grade characteristics, and can be considered the same lesion. Hence, additional cases are required to identify the inherent nature of such soft tissue tumors harboring *ALK* rearrangements and standardize their categorization.

In this study, we analyzed two IFS tumors with *ALK* gene rearrangements in children and summarized the clinicopathological characteristics of these kinase fusion-positive mesenchymal neoplasms, hoping to identify new approaches.

## Case presentation

### Clinicopathological findings

**Figure 1:**
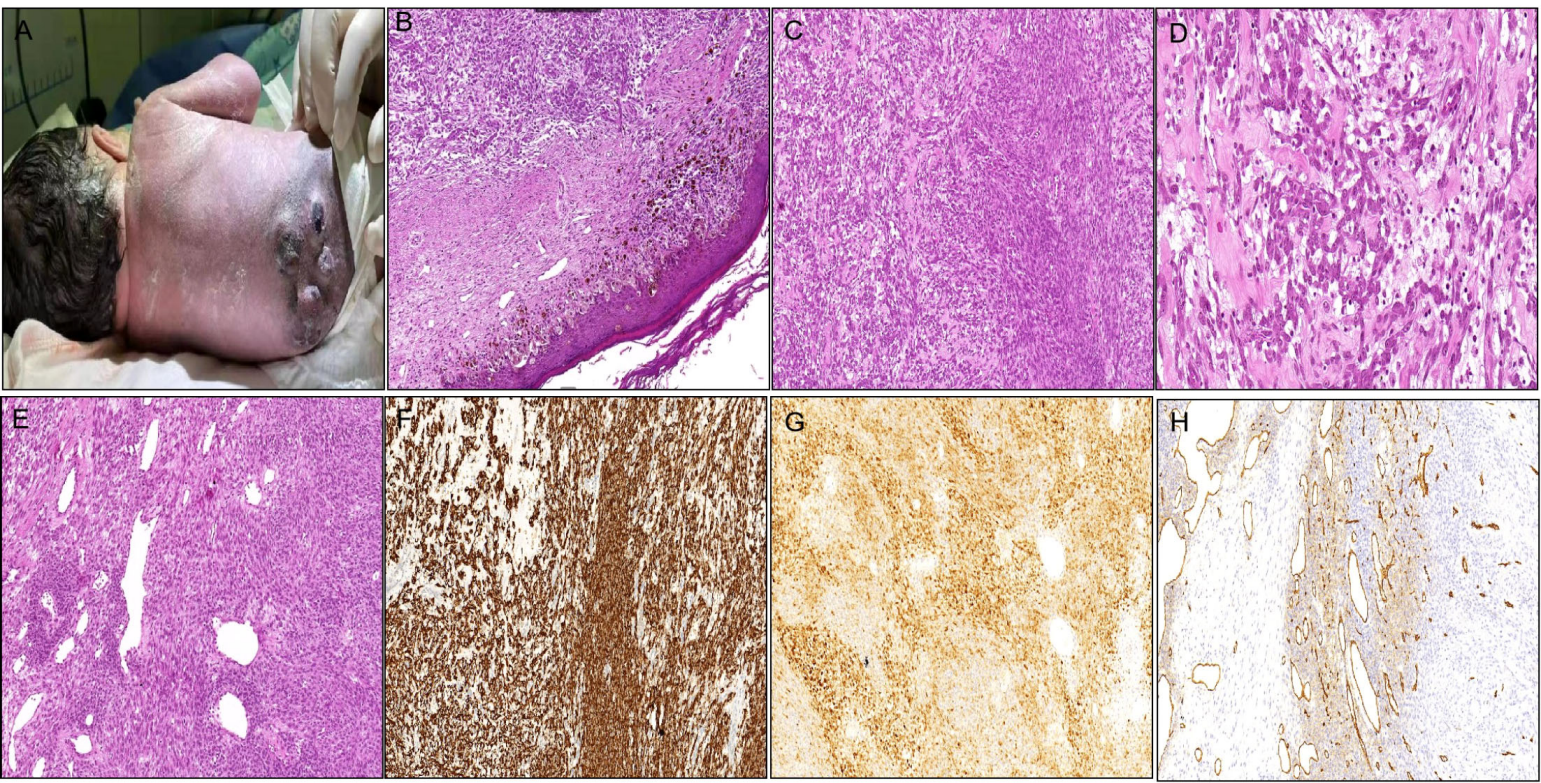
Clinicopathological findings of the tumor in Case 1. **(A)** Multiple subcutaneous nodules on the lumbar back with small black spots in the surrounding skin. **(B)** Nevus cell clusters in the epidermis and superficial dermis [**(B)** H&E staining×200]. **(C)** Spindle to epithelioid cells in long fascicles or haphazardly in myxoid-to-collagenous stroma [**(C)** H&E staining×200]. **(D)** Some ovoid tumor cells mixed with mild infiltrating inflammatory cells [**(D)** H&E staining×400]. **(E)** The tumor periphery demonstrating HPC-like vasculature [**(E)** H&E staining×200]. **(F)** Tumor cells densely cytoplasmic positive for ALK-D5F3. **(G)** Tumor cells densely positive for S100. **(H)** Tumor cells focally positive for CD34.

Case 1 was a 6-day-old male newborn who presented with multiple subcutaneous nodules in the lumbar back region, which were first detected at 30 weeks of gestation by trimester ultrasound. The pregnancy was otherwise uncomplicated, aside from fetal distress necessitating cesarean delivery at 35 weeks. Gross examination at birth revealed multiple subcutaneous nodules on his lumbar back with dense little black spots in the surrounding skin (**Figure 1A**). The clinical presentation and imaging characteristics indicated that the initial clinical impression was congenital nevus or neurogenic tumor. The largest mass (2 cm × 2 cm × 1 cm) was resected and part of the surface was ulcerated. Microscopic examination revealed congenital melanocytic nevi in the epidermis and superficial dermis with no obvious mitosis in the epidermal ulceration area (**Figure 1B**). The subcutaneous adipose tissue and dermis contained a nodular lesion, characterized by spindle to epithelioid cells lacking melanin arranged in long fascicles or randomly in a myxoid-to-collagenous stroma (**Figure 1C**). Furthermore, some ovoid tumor cells mixed with slight infiltrating inflammatory cells (**Figure 1D**), closely resembling an epithelioid inflammatory myofibroblastic tumors (IMT). Some epithelioid cells presented plentiful cytoplasm and small nucleoli. The tumor periphery exhibited hemangiopericytoma (HPC)-like vasculature (**Figure 1E**). The cellular region contained abundant mitotic figures (MFs), with approximately 3–8 MFs observed per 10 high-power fields (HPFs). Based on the grading system of Fédération Nationale des Centres de Lutte Contre le Cancer (FNCLCC) (8), the tumor exhibited low-grade features, corresponding to grade 1, with a differentiation score of 2, a mitotic count score of 1, and a necrosis score of 0. Immunohistochemistry demonstrated that the superficial nevus cells expressed S100, SOX10, and melanocytic markers (HMB45, Melan-A, and MiTF), while they were negative for ALK-D5F3 and CD34. In contrast, the deep neoplastic cells revealed strong positivity for ALK-D5F3 (**Figure 1F**) and S100 (**Figure 1G**), exhibited partial positivity for desmin and CD34 (**Figure 1H**), and were negative for SOX10, HMB45, Melan-A, MiTF, MyOD1, myogenin, SMA, Actin, pan-TRK, BRAF-V600E, and CK. The average Ki-67 index was 30%. The parents of the baby rejected therapy after the pathological diagnosis. There were no signs of progression or metastasis with limited follow-up information (36 months).

**Figure 2:**
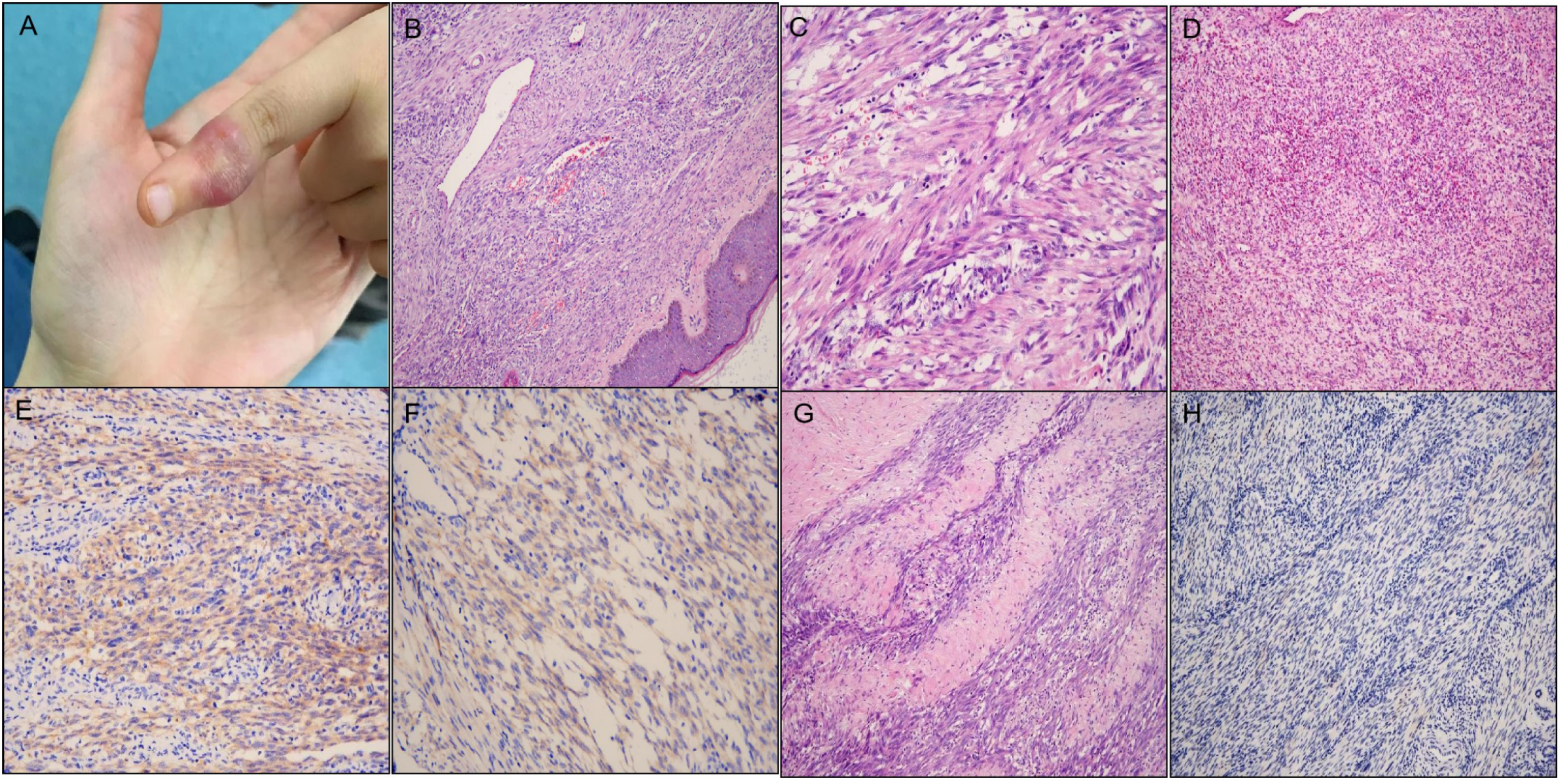
Clinicopathological findings of the tumor in Case 2. **(A)** A subcutaneous mass on the left index finger. **(B)** Microvascular proliferation and focal ectatic vessels [**(B)** H&E staining×200]. **(C)** Spindle cells arranged in a fascicular growth or haphazard pattern with indistinct cytoplasmic borders and mild to moderate nuclear pleomorphism [**(C)** H&E staining×400]. **(D)** Moderate inflammatory cell infiltration [**(D)** H&E staining×100]. **(E)** Tumor cells of the finger immunohistochemically cytoplasmic positive for ALK-D5F3. **(F)** Tumor cells of the finger immunohistochemically positive for CD34. **(G)** Metastasized tumor demonstrating similar morphology to the original tumor, with more compact tumor cells interspersed in collagen matrix [**(G)** H&E staining×200]. **(H)** The tumor cells of the left axilla were immunohistochemically negative for ALK-D5F3.

Case 2 was a 9-year-old girl with a mass on her left index finger (**Figure 2A**). Under gross examination, the biopsy specimen consisted of a 0.6 cm × 0.4 cm × 0.3 cm skin fragment. Microscopically, the lesion consisted of spindle and ovoid cells infiltrating the fat tissue, and microvascular proliferation and focal ectatic vessels (**Figure 2B**). The spindle cells were organized in a fascicular or random pattern, with indistinct cytoplasmic borders and mild to moderate-variability nuclear pleomorphism (**Figure 2C**). Furthermore, moderate inflammatory cell infiltration (**Figure 2D**) and brisk mitosis were observed (~5–6 MFs/10 HPFs). FNCLCC grading corresponded to histologic grade 1, with a differentiation score of 2, a mitotic count score of 1, and a necrosis score of 0. Immunohistochemical staining of the tumor cells in the finger was positive for ALK-D5F3 (**Figure 2E**), S100, CD34 (**Figure 2F**) and negative for AE1/3, SMA, desmin, STAT6, SOX10, and pan-TRK. A 96-month clinical follow-up revealed that the girl experienced double recurrences and required partial finger amputation due to metastases to the left axilla. Initially, the girl had a small red nodule, resembling a millet seed, protruding from the skin of the left index finger. This was not considered significant at the time. Two years later, as the nodule increased in size, the patient underwent cryotherapy supplemented with topical corticosteroid ointment. However, there was no improvement. A biopsy of the mass was then performed, and the pathology revealed infantile cellular hemangioma. Subsequently, the patient underwent surgical resection of the mass with negative postoperative margins. The pathology suggested a soft tissue tumor, but the specific diagnosis remained unclear. Four months after the surgery, the tumor recurred, prompting another resection. The pathology was reviewed by multiple hospitals, but opinions varied, and a definitive diagnosis could not be established. No further treatment was administered after this surgery. Four years later, the tumor recurred once more, and a metastatic lesion was discovered in the left axilla. The patient then underwent partial finger amputation and excision of the metastatic lesion. As of the latest follow-up, the patient remained alive with no recurrence. Pathologically, the relapsed and metastasized tumor exhibited similar morphology to the original tumor and was characterized by more compact tumor cells interspersed in collagen matrix (**Figure 2G**). Surprisingly, the tumor cells of the left axilla were immunohistochemically negative for ALK-D5F3 (**Figure 2H**), which differed from the primary tumor. The other immunophenotype of the metastasized tumor was similar to that of the original tumor.

### Next-generation sequencing targeted genomic profiling

Genomic RNA was extracted from formaldehyde-fixed paraffin-embedded (FFPE) tumor tissues using magnetic beads. RNA was reverse-transcribed to complementary DNA using reverse transcriptase. Because the case series were from two regional hospitals, the NGS of the two patients involved two different approaches, panels, and sequencing companies. For Case 1, an AmpliSeq RNA SARC Fusion panel consisting of 204 primer pairs specific for 64 fusion gene pairs and primers specific for 10 internal reference genes was used to amplify soft tissue tumor-associated fusion genes and build amplicons. Sequencing was performed using an Ion PGM system kit (conducted by KingMed Center, Guangzhou, China).

For Case 2, comprehensive gene sequencing was conducted using a capture-based targeted sequencing panel (provided by Geneseeq Biotech, Nanjing, China) to detect RNA-based fusions and other genomic alterations. This panel included 506 genes and was capable of identifying single base substitutions, short and long insertions/deletions, copy number variations, gene fusions, and rearrangements.

**Figure 3:**
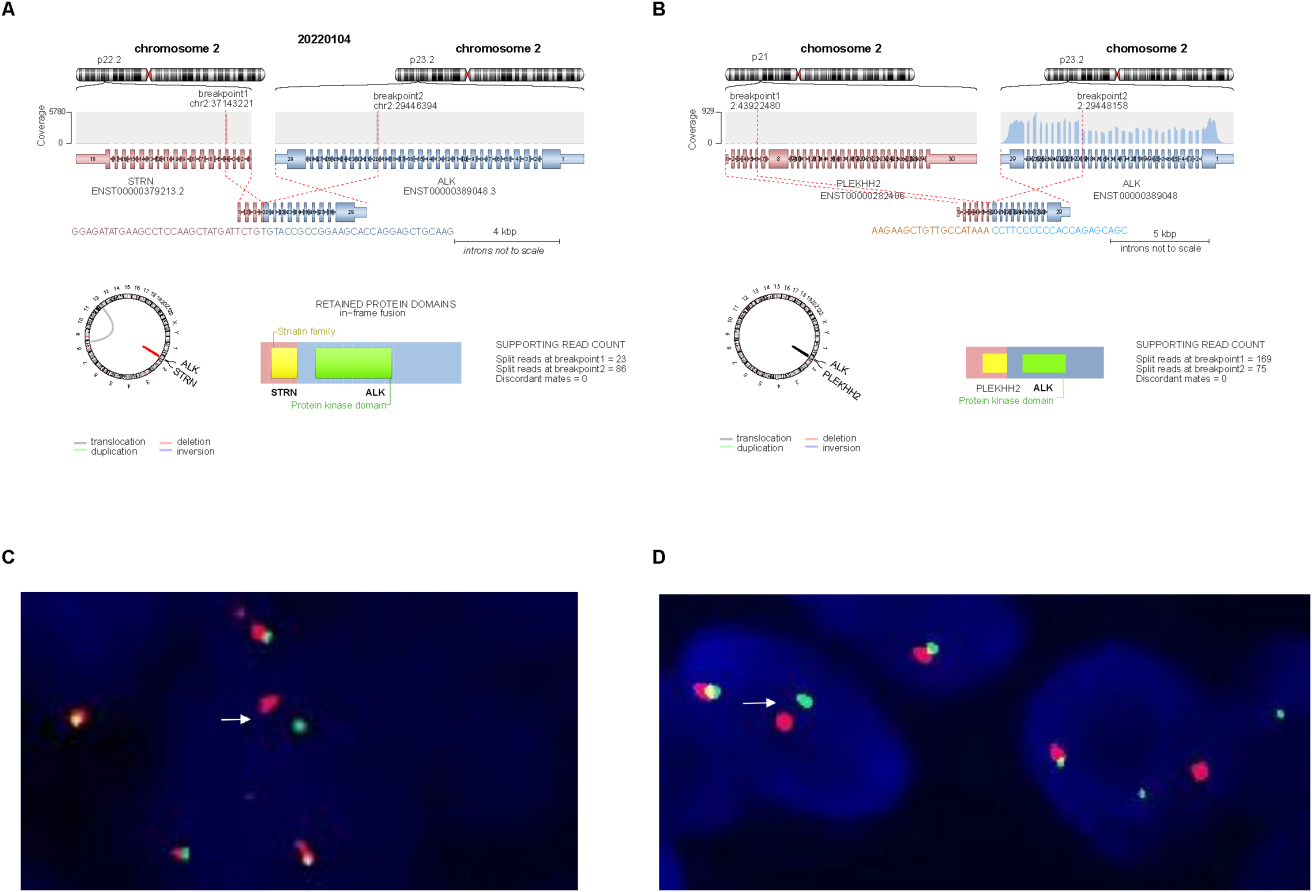
Molecular findings. **(A)** NGS identified a transcript comprising *STRN* exon 3 and *ALK* exon 20 in Case 1. **(B)** NGS identified a transcript comprising *PLEKHH2* exon 6 and *ALK* exon 20 in Case 2. **(C, D)** *ALK* break-apart FISH test was positive in Case 1 **(C)** and Case 2 **(D)**, demonstrating a signal pattern consisting of isolated 5′ (green), isolated 3′ (red), and fused 3′/5′ signals.

NGS of Case 1 revealed a fusion transcript between *STRN* exon 3 and *ALK* exon 20 (**Figure 3A**), while Case 2 carried a fusion transcript between *PLEKHH2* exon 6 and *ALK* exon 20 (**Figure 3B**). No additional pathogenic variants were identified in both cases. The predicted chimeric proteins comprised a coiled-coil domain in the *STRN* or *PLEKHH2* N-terminus and a complete kinase domain in the *ALK* C-terminus.

### Fluorescence *in situ* hybridization

FISH was performed on 3-µm thick FFPE tumor sections using an ALK dual-color break-apart probe (Vysis, Abbott Molecular). More than 15% of the tumor cells with abnormal signals were considered positive for gene rearrangement based on the scoring of 100 non-overlapping cells. FISH confirmed that both cases carried *ALK* gene rearrangements (**Figures 3C, D**).

## Discussion

We describe two pediatric *ALK*-rearranged spindle tumors with significant clinicopathological similarities to IFS. Histologically, both cases exhibited typical IFS morphology, including spindle to oval cells arranged either in long fascicles or haphazardly within a myxoid-to-collagenous stroma. Case 1 also exhibited HPC-like vessels. The immunohistochemistry of both cases demonstrated cytoplasmic ALK expression, with partly co-expressed CD34 and S100. This staining pattern is most commonly reported in *NTRK*-rearranged spindle cell neoplasms (9), whereas CD34 and/or S100 expression has been less frequently reported in IFS.

*ALK* rearrangements in pediatric spindle cell neoplasms often suggest the diagnosis of IMT (10), but the tumors in this study displayed clinicopathological features more consistent with IFS. Unlike IMTs, the two cases did not demonstrate significant lymphoplasmacytic or eosinophilic inflammation, nor contained ganglion-like mesenchymal cells. Furthermore, IMTs typically arise in the body cavities, lungs, soft tissues, and viscera of young patients (11, 12), with cutaneous IMTs being exceptionally rare. IMTs are fibroblastic/myofibroblastic tumors with strong SMA expression, with approximately 50–60% of cases carrying *ALK* gene fusions (11). Various *ALK* gene partners have been reported in IMTs, such as *A2M*, *ATIC*, *CARS*, *CLTC*, *DCTN1*, *DES*, *EML4*, *FN1*, *HNRNPA1*, *IGFBP5*, *LMNA*, *PPFIBP1*, *PRKAR1A*, *RANBP2*, *RRBP1*, *SEC31L1*, *TFG*, *THBS1*, *TIMP3*, *TNS1*, *TPM3*, and *TPM4* (12, 13). The *ALK::STRN* fusions (14) and *ALK::PLEKHH2* fusions (15) in the present study have also been described in IMT, but it is rare for these fusions to occur in cutaneous IFS-like tumors.

Interestingly, the clinical presentation and H&E-stained morphology led us to exclude a diagnosis of melanocytic tumors, such as melanomas arising in congenital naevi, atypical Spitzoid melanocytic tumor or Spitzoid melanoma with *ALK* fusion, Melanocytic myxoid spindle cell tumor with *ALK* rearrangement (MMySTAR) in case 1. Immunohistochemistry revealed the absence of SOX10 and melanocytic markers in the deep neoplastic cells, suggesting that these cells were non-melanocytic. Furthermore, we performed NGS testing, which covered a comprehensive panel of melanoma-associated molecular markers, including *BRAF, NRAS, HRAS, CCND1, RET, KIT, CDKN2A*, and the *TERT* promoter. However, no mutations were detected, further substantiating the absence of melanoma-related malignancies.

We detected two *ALK* fusions, each with a unique fusion partner, including one fusion (Case 1: *STRN*::*ALK*) and another fusion gene (Case 2: *PLEKHH2*::*ALK*). *STRN* without its coiled-coil domain or *ALK* with a tyrosine kinase domain mutation results in the absence of protein expression, and *ALK* with a tyrosine kinase sequence mutation does not lead to carcinogenesis (16). To the best of our knowledge, 46 cancer cases carried *STRN* exon 3 to *ALK* exon 20 fusion have been reported (17), including the present case, which comprises 3 malignant peritoneal mesothelioma cases, 31 cases of thyroid cancer, 5 cases of lung cancer, 3 cases of colorectal cancer, 2 cases of renal cancer, and 1 case of pancreatic cancer. *PLEKHH2* encodes an intracellular protein highly enriched in renal glomerular podocytes and supports the podocyte foot processes (18). The N-terminus of PLEKHH2 contained a putative ahelical coiled-coil domain. *PLEKHH2*::*ALK* gene fusion has been reported in lung adenocarcinoma (19) and dermatofibrosarcoma protuberans (DFSP) (20). The positive response to ALK inhibitors in lung tumors with *PLEKHH2::ALK* fusion proteins further confirms their oncogenic potential. Commonly, *ALK* fusions activate the ALK kinase domain through autophosphorylation resulting from dimerization without requiring ligands (21). The fusion genes in this study contained the entire *ALK* intracellular kinase domain and the coiled-coil domain of the fusion partner genes, which mediated ALK dimerization and activation. Accordingly, we assumed that the fusion proteins were oncogenic.

In the second case, the metastatic lesion in the left axilla exhibited a loss of ALK expression on immunohistochemical analysis, while other immunophenotypic features remained consistent with those of the primary lesion. However, both lesions demonstrated *ALK* rearrangements with the same fusion partner, *PLEKHH2*, in NGS testing. The mutation abundance was 59.74% in the primary lesion and 42.47% in the metastatic lesion, and the tumor mutation burden (TMB) was 0 in both lesions. We hypothesize that this discrepancy may be due to the following potential mechanisms: First, metastatic clones may gain selective advantages by suppressing ALK expression through epigenetic modifications or other mechanisms, and may activate alternative signaling pathways to compensate for the loss of *ALK* signaling. Second, the microenvironment of the metastatic site, influenced by factors such as immune cell infiltration, cytokines, or hypoxia, may differ from that of the primary tumor and affect ALK expression. Third, metastatic cells may exhibit altered transcriptional regulation or RNA stability, with changes in transcription factors or miRNAs targeting ALK mRNA, leading to reduced ALK protein expression. Fourth, metastatic cells may enhance ALK protein degradation via the ubiquitin-proteasome pathway or inactivate ALK through dephosphorylation. Fifth, tumor heterogeneity and sampling bias in the metastatic lesion might result in the analysis of areas with lower ALK expression. Finally, epigenetic modifications, such as DNA methylation and histone modification, may silence *ALK* gene expression despite the presence of *ALK* rearrangements.

*ALK* fusion-driven soft tissue tumors exhibit varying specific histologic features across a broad histopathological spectrum (15). These neoplasms range from low- to intermediate-grade and are characterized by different underlying kinase fusions, which may demonstrate a pattern resembling the lipofibromatosis-like neural tumor (22) or IFS phenotype (5), or may resemble malignant peripheral nerve sheath tumors (23) and frequently contain regions with stromal and/or perivascular hyalinization (7). A small minority of kinase-fused neoplasms may exhibit malignant characteristics such as high cellularity, diffuse hyperchromasia, increased mitotic activity, and necrosis, leading to aggressive clinical behavior with distant metastases and fatal outcomes. Mesenchymal neoplasms with kinase fusion consistently exhibit cytologic monotony, regardless of where they are on this spectrum.

Despite their diagnostic terminology variations, IMT and IFS demonstrate similar outcomes, with a 25% recurrence risk and low rates of distant metastatic disease (12, 24). One of the most striking aspects of these pediatric kinase-rearranged mesenchymal tumors is the disconnect between traditional histologic grade based on FNCLCC and clinical outcome. As illustrated by Case 2, which is “low-grade” by FNCLCC, but the patient developed rapid recurrence and metastasis. This phenomenon may be caused by a variety of factors, including the genetic characteristics of the tumor, the microenvironment, and individual differences among patients. Reports on this aspect are scarce, we currently lack reliable prognostic markers for this category of tumors and need further research to understand their long-term clinical outcomes. Notably, *ALK* fusions in IFS-like tumors indicate a positive response to ALK inhibitor treatment, such as crizotinib. Furthermore, targeted therapeutics reduce mortality from aggressive tumors and alleviate morbidity from indolent tumors in challenging anatomical locations. Unfortunately, neither of our two cases was treated with ALK inhibitors. Particularly in Case 2, because the initial pathological diagnoses were inconclusive, effective treatment was not initiated, leading to multiple recurrences and ultimately necessitating amputation of the finger. Had we more accurately recognized the nature of such tumor and initiated early diagnosis and treatment, especially with ALK inhibitors, amputation might have been avoided.

Tan SY et al (5). described a series of four pediatric *ALK*-rearranged mesenchymal spindle cell tumors that exhibited clinicopathological features similar to IFS. Two tumors originated in the kidney, while the other two developed in soft tissues. Histologically, all cases demonstrated a morphological spectrum typical of IFS, characterized by cellular, spindle to ovoid cells arranged either in long fascicles or haphazardly within a collagenized to myxoid stroma. These tumors also featured HPC-like vessels and focal perivascular hyalinosis. *ALK* fusions were identified in all four tumors, each with a unique fusion partner. One renal tumor showed co-expression of S100 and CD34 and developed liver and lung metastases 1–2 months after presentation. On this basis, we have additionally reported two cases that share similar morphological, immunohistochemical, molecular, and clinical features with the previously described case series. This expanded case series highlights the considerable genetic overlap among inflammatory myofibroblastic tumors (IMT), cellular congenital mesoblastic nephroma (cCMN)/IFS, and *NTRK*-rearranged spindle cell neoplasms. These findings suggest that these entities may represent a continuum of kinase-related mesenchymal tumors or distinct but morphologically, immunophenotypically, and genetically overlapping groups of tumors. This insight underscores the potential for a more refined reclassification in the future.

## Conclusion

Herein, we report two additional cases of *ALK*-rearranged mesenchymal neoplasms, each exhibiting distinct characteristics that support the overlap between IFS-like tumors and *NTRK*-rearranged spindle cell neoplasms. These findings suggest that *ALK* is involved in the development of these tumors. Identifying spindle cell tumors within this spectrum may have significant therapeutic implications.

## Data Availability

The original contributions presented in the study are included in the article/supplementary material. Further inquiries can be directed to the corresponding authors.
